# Prehospital airway and operational management of severe ammonia intoxication: a case report

**DOI:** 10.1186/s12245-026-01308-9

**Published:** 2026-07-16

**Authors:** Lydia Johnson Kolaparambil Varghese, Regina Schulz, Raphael Abels, Gerrit Jansen

**Affiliations:** 1 Johannes Wesling Hospital Minden, Department of Anaesthesiology, Intensive Care Medicine, Emergency Medicine and Pain Management, Ruhr University Bochum, University, Hans-Nolte-Straße 1, 32429 Minden, Germany; 2 Medical Faculty of Ruhr University Bochum, Ruhr University Bochum, Universitätstraße 150, 44801 Bochum, Germany

**Keywords:** Ammonia, Airway management, Inhalation, Burns, Chemical, Hazardous substances, Emergency medical services

## Abstract

**Background:**

Ammonia inhalation can cause rapid airway compromise and noncardiogenic pulmonary oedema. Although hospital management has been described, prehospital strategies in severe cases remain largely undocumented.

**Case presentation:**

A 44-year-old obese male (160 kg) was exposed to an ammonia cloud at a decommissioned power plant. He presented with mucosal burns, severe dyspnoea, and hypoxemia despite high-flow oxygen. Prehospital providers initiated nebulized adrenaline, performed ocular irrigation, and prepared for surgical airway backup. Rapid sequence intubation was conducted under video laryngoscopy. Immediately post-intubation, copious foamy secretions confirmed fulminant pulmonary oedema. Despite FiO₂ 1.0 and a PEEP of 12 cmH₂O, oxygenation remained marginal at 89–90%. Intravenous corticosteroids were administered, burns were covered, and the patient was transported by helicopter to a burn and critical care centre. On-scene decontamination was deferred due to instability; the EMS team underwent full decontamination after patient handover. Ground transport to the nearest regional centre would have required approximately 60 min; HEMS transport directly to the tertiary burn centre (MHH Hannover) took approximately 35 min. Beyond transport time, HEMS use involved inherent aeromedical risks: residual ammonia off gassing in the confined cockpit and cabin posed a risk of mucosal irritation or pilot incapacitation, and inadvertent chemical contamination of sensitive avionics or airframe components posed a corrosion risk. These were weighed against clinical urgency and the absence of adequate regional ICU or burn capacity. No formal risk-based framework currently exists to guide HEMS transport of CBRN-exposed patients; this case underscores the need for one.

**Conclusions:**

This case demonstrates the unique prehospital challenges of ammonia inhalation: the need for early definitive airway management, refractory hypoxemia beyond the capacity of prehospital ventilators, and operational dilemmas such as delayed decontamination, patient weight, and limited local ICU resources. It highlights the urgent need for structured EMS protocols for caustic inhalation events. Beyond the clinical case, this report addresses the complexity of transport decision-making in critically ill patients with incomplete decontamination, including civil aviation safety considerations and the absence of a risk-based framework for CBRN aeromedical transport.

## Background

Ammonia is one of the most widely used industrial chemicals worldwide, with major applications in refrigeration, agriculture, and energy production. Exposure to high concentrations can result in severe alkali injury to mucous membranes, upper airway burns, bronchospasm, and rapid progression to noncardiogenic pulmonary oedema and acute respiratory distress syndrome (ARDS) [1, 2]. Mortality is primarily related to airway compromise and hypoxemia.

Although case series of ammonia intoxication exist in toxicology and burn literature [3–5], most describe hospital management. In contrast, little is known about prehospital strategies when patients present with imminent airway obstruction and refractory hypoxaemia. Furthermore, few reports discuss the operational dilemmas that confront emergency medical services (EMS) when facing hazardous material incidents in resource-limited environments.

We present a case of severe ammonia intoxication with rapid prehospital airway deterioration, complicated by pulmonary oedema, morbid obesity, and system-level constraints. The case illustrates clinical, toxicological, and logistical challenges and contributes to the limited literature on prehospital management of caustic gas inhalation.

## Case presentation

At 13:28, EMS was dispatched to a decommissioned power plant with the call “ammonia cloud, inhalation trauma, severe dyspnoea.” En route, dispatch reported that the hazard had been contained and no ongoing risk to the public was present. Given the anticipated severity helicopter transport to a tertiary burn and critical care centre was already requested while en route to the scene.

Upon arrival, a 44-year-old obese male (160 kg) was found outdoors, unclothed, after attempting self-decontamination. He was in obvious respiratory distress and had been placed on high-flow oxygen by first responders. Examination revealed mucosal burns to the oral cavity and pharynx, suggesting impending airway compromise. Respiratory rate was 30 breaths per minute with oxygen saturation of 95% on 15 L/min oxygen. He was tachycardic at 130 beats per minute, blood pressure was 140/80 mmHg, and skin was cool and clammy, but perfusion remained adequate. He was alert and oriented with no neurologic deficit. Approximately 15% of total body surface area exhibited partial-thickness burns, involving the right thigh, abdomen, scrotum, and right chest and arm. The incident occurred in February in the Kreis Minden-Lübbecke region of north-western Germany, with ambient temperatures of approximately 2 °C (mid-winter); the patient had been unclothed outdoors for passive off-gassing, creating a substantial risk of hypothermia in addition to inhalation injury.

Given the imminent threat of airway obstruction, rapid sequence intubation (RSI) was undertaken. The patient received nebulized adrenaline and ocular irrigation prior to induction. A peripheral intravenous line was established, and a cricothyrotomy set was prepared. RSI was performed with fentanyl 0.5 mg, propofol 300 mg, and rocuronium 100 mg. Video laryngoscopy enabled atraumatic intubation without difficulty. Although the patient had removed his clothing, no structured decontamination had occurred. During induction, he remarked, ‘I have not yet been washed,’ emphasizing the uncertainty of his decontamination status. Given critical respiratory compromise, definitive airway management was prioritized over complete decontamination. Induction agent selection reflected individual practitioner judgment; the shock index of 1.0 was noted but perfusion remained adequate. Ketamine is an appropriate alternative in haemodynamically compromised patients and is increasingly adopted globally in prehospital RSI. Fentanyl dosing was weight-based per local protocol; the relevance of lean body weight dosing and the association between fentanyl and non-cardiogenic pulmonary oedema (NCPE) warrant consideration in patients with compromised pulmonary vasculature. Decontamination was deferred, not abandoned - it was completed after patient handover, as soon as clinically feasible.

Immediately post-intubation, copious foamy secretions were observed, confirming acute pulmonary oedema. Mechanical ventilation was initiated with FiO₂ 1.0 and a PEEP of 12 cmH₂O, maintaining oxygen saturation only at 89–90%. Intravenous prednisolone 250 mg was administered. Two additional intravenous lines were secured, and chemical burns were dressed.

Due to persistent hypoxemia and the absence of local intensive care capacity, helicopter transfer to a burn and critical care centre was prioritized over on-scene decontamination. Despite logistical difficulties posed by the patient’s body habitus, transport was successful. The EMS team underwent full decontamination after patient handover.

In Germany, EMS is physician-staffed, and advanced airway interventions including rapid sequence intubation are available in the prehospital setting. In Kreis Minden-Lübbecke, prehospital resources include paramedic teams, physician-staffed response units, and helicopter transfer capability. Despite this, regional critical care and burn capacity is limited, often requiring transfer to tertiary centres. On arrival at the tertiary burn and critical care centre (Medical School Hannover Hospital, MHH), the patient was admitted to the intensive burn care unit. He received advanced ventilatory support and burn management. He required prolonged mechanical ventilation and tracheotomy. After stabilization, he was transferred to a specialized weaning facility. He survived with a favourable prognosis.

## Discussion and conclusions

This case highlights the rapid clinical deterioration and operational complexity associated with prehospital ammonia intoxication.

Ammonia is a water-soluble alkaline gas that penetrates moist mucosal surfaces, producing liquefactive necrosis [1, 6]. Severe inhalation leads to airway oedema, bronchospasm, and pulmonary oedema, which may progress to ARDS [7–9].

Nebulized adrenaline was administered prior to induction to reduce supraglottic oedema and facilitate laryngoscopy; nebulization via an endotracheal tube post-intubation is not indicated in this context. Its pre-intubation use remains a pragmatic but evidence-limited strategy, derived mainly from smoke inhalation experience This reflected anticipation of a “triple difficult airway”: physically (morbid obesity BMI∸50, oropharyngeal mucosal burns, risk of supraglottic oedema); physiologically (pre-intubation hypoxaemia limiting safe apnoeic time); and from a provider-safety perspective (ammonia exposure risk limiting proximity and duration of airway assessment) [10, 11].

In this case the Weinmann MEDUMAT Standard² was used, which does not support inverse ratio ventilation or active recruitment manoeuvres. Systems with more advanced capabilities (e.g. Hamilton T1) exist in some EMS configurations, so ventilator limitations are context-specific. Regardless, refractory hypoxaemia unresponsive to available settings underscores the importance of rapid transfer to a tertiary centre with extracorporeal support capability [3].

Pharmacologic adjuncts. Corticosteroid therapy in chemical inhalation injury remains controversial. While some studies suggest no clear benefit, steroids are frequently administered in practice with the aim of reducing airway oedema [12, 13]. In this case, prednisolone was given empirically, reflecting common EMS practice in the absence of robust evidence. Importantly, once the airway was secured, upper airway oedema was no longer the primary concern. The rationale for corticosteroid administration more accurately reflects an attempt to attenuate chemical pneumonitis—the direct lower airway and alveolar injury caused by ammonia’s alkaline properties—and ongoing mucosal inflammation, rather than upper airway oedema per se.

The case also underscores the broader operational dilemmas of hazardous material incidents and the difficulty of verifying decontamination status in unstable patients. Initial reports suggested ongoing risk, later downgraded. The patient was incompletely decontaminated, raising concerns for crew safety, yet full on-scene decontamination was deferred due to clinical instability. Chemical incidents often require undressing for off gassing, but in cold environments this introduces the additional hazard of hypothermia. In this case, February temperatures and outdoor exposure compounded the patient’s critical status. Patient obesity complicated helicopter transfer. While guidelines recommend decontamination prior to treatment, patient survival may necessitate reversal of this sequence. Such decisions underscore the tension between patient stabilization and provider safety during hazardous material incidents. These realities highlight the need for structured EMS protocols that balance patient stabilization with crew protection and system limitations. Transporting an incompletely decontaminated patient by HEMS carries specific risks for the aeromedical crew: residual ammonia off-gassing in the confined cockpit and cabin may cause mucosal irritation or, at higher concentrations, pilot incapacitation—a potentially catastrophic outcome in flight. Aeromedical platforms lack a decontamination corridor, and inadvertent chemical contamination of avionics or structural components poses a corrosion risk largely absent in ground transport. These hazards must be explicitly incorporated into CBRN aeromedical transport risk assessments and crew safety protocols.

Survival following fulminant ammonia inhalation injury depends on rapid airway management and specialized intensive care. In this case, timely prehospital RSI, prioritization of transport, and access to a tertiary burn centre contributed to a favourable outcome, underscoring the importance of structured EMS response and regionalized care pathways.

Beyond caustic inhalation, this case highlights the paucity of evidence and consensus surrounding transport decision-making for critically ill patients exposed to chemical, biological, radiological and nuclear (CBRN) materials. The operational dilemmas encountered—deferred decontamination, aeromedical transport of a volatile hazard, and incomplete crew protection protocols—are not unique to ammonia. Structured, risk-based EMS frameworks addressing these challenges across the CBRN spectrum are urgently needed and represent an important direction for future research and guideline development.

## Data Availability

No datasets were generated or analysed during the current study.

## Abbreviations

ARDS: Acute respiratory distress syndrome
EMS: Emergency medical services
FiO₂: Fraction of inspired oxygen
ICU: Intensive care unit
PEEP: Positive end-expiratory pressure
RSI: Rapid sequence intubation
